# Comparative transcriptomics reveal contrasting strategies between a fungal plant pathogen and an endophyte during initial host colonization

**DOI:** 10.1128/spectrum.00226-25

**Published:** 2025-06-12

**Authors:** Soumya Moonjely, Frances Trail

**Affiliations:** 1 Department of Plant Biology, Michigan State University 124535 , East Lansing, Michigan, USA; 2 Department of Plant, Soil and Microbial Sciences, Michigan State University 242456 , East Lansing, Michigan, USA; Universita degli Studi del Molise, Campobasso, Italy

**Keywords:** *Fusarium graminearum*, *Metarhizium anisopliae*, conidial germination, RNA sequencing, plant host colonization, CAZymes, specialized metabolites, effector proteins

## Abstract

Conidial germination marks the beginning of the fungal life cycle on the host plant, leading to disease or mutually beneficial relationships. Using comparative transcriptomics, we aim to unravel the transcriptional similarities and differences of *Fusarium graminearum* (plant pathogen)*,* and *Metarhizium anisopliae* (endophyte), during conidial germination and initial colony establishment, to identify the key traits that support their distinct lifestyles. *F. graminearum* and *M. anisopliae* belong in the order, *Hypocreales*. However, their contrasting roles as pathogen and endophyte provide an excellent model for exploring initial colony establishment on the hosts. Our comparison crosses four stages including fresh conidia, polar growth, hyphal extension, and first hyphal branching (on medium) or appressorium formation (on barley). *F. graminearum* exhibited a higher number of upregulated genes associated with host interactions, including genes for CAZymes, specialized metabolites, and effectors, particularly during the appressorium stage, reflecting its pathogenic nature. By comparison, analysis of the *M. anisopliae* transcriptome revealed reduced transcript levels of CAZyme and specialized metabolite genes, reflecting a less aggressive host penetration approach. The candidate genes associated with indole-3-acetic acid synthesis were upregulated during the appressorium stage in *M. anisopliae*, supporting its endophytic lifestyle, and suggesting that the fungus uses a phytohormone-based strategy to interact with plant hosts. Collectively, our findings provide valuable insights into the gene networks directing conidial germination and initiation of infection in pathogenic versus endophytic fungi, as well as documenting appressorium formation of *M. anisopliae* for the first time in barley.

**IMPORTANCE:**

Conidial germination is the initial step for fungal colonization in diverse environments. Here, we examine the transcriptional similarities and differences in conidial germination and colony establishment of *Fusarium graminearum* and *Metarhizium anisopliae*, two fungal species with distinct lifestyles belonging to the Order Hypocreales. *F. graminearum* is a plant pathogen and the causal agent of Fusarium head blight on cereal crops, whereas *M. anisopliae* is an insect pathogen and root endophyte which forms beneficial associations with plants. We compared the transcriptome profiles of these species under two nutrient conditions across four developmental stages of conidial germination. Our study shows that the expression profile of genes encoding carbohydrate-active enzymes, specialized metabolites, and putative effectors varies between *F. graminearum* and *M. anisopliae*. The results of this study provide insights into gene networks associated with spore germination stages on the host in a pathogenic vs an endophytic fungus.

## INTRODUCTION

**Fig 1:**
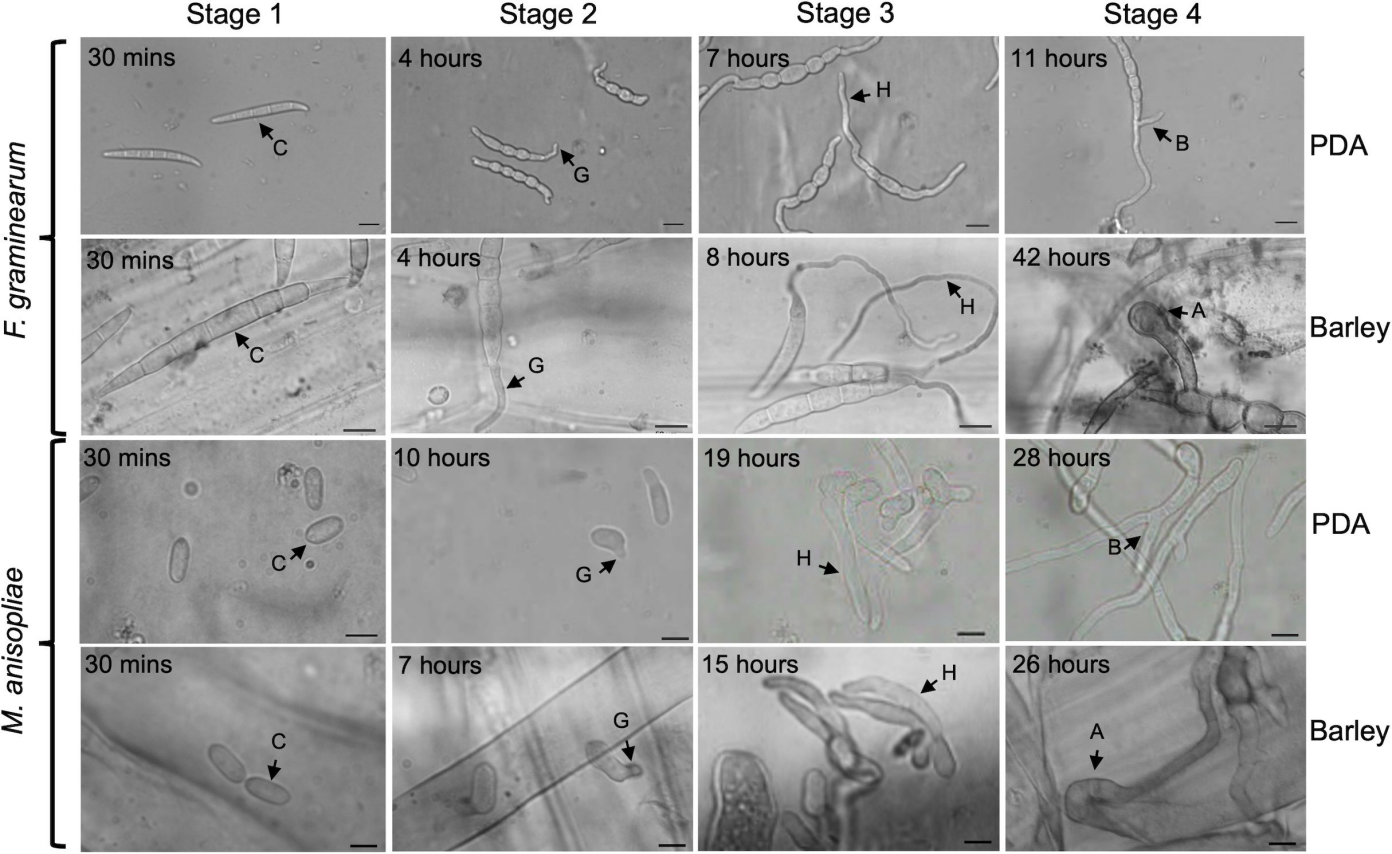
Time course and morphology of conidial germination stages in *F. graminearum* and *M. anisopliae* under two growth conditions on media and on barley. C, conidium; G, germ tube; H, hypha; B, hyphal branching; A, appressorium. Scale bars: 20 µm (*F. graminearum*); 5 µm (*M. anisopliae*). Stage 1: Fresh conidia; Stage 2: Polar growth; Stage 3: Hyphal extension; Stage 4: First hyphal branching (PDA) or appressorium (barley).

Conidial germination is an important step in the life cycle of many fungal species for growth, survival, and reproduction. It is also a key step in fungal pathogenesis as the initial adhesion of the fungal conidia and subsequent steps, including germination and infection structure formation, are critical for the persistence of fungi in the host (1). After dispersal of conidia into the environment, germination marks the initiation of fungal growth to establish new colonies. During germination (Fig. 1), conidia break dormancy (Stage 1) through isotropic swelling to initiate metabolic activities (2–4); begin polar growth (Stage 2), characterized by the emergence of the germ tube; proceed through hyphal extension (Stage 3); and, depending on the substrate, extend hyphal growth through branching (Stage 4) or forming appressoria (Stage 4), specialized infection structures. Appressoria breach the physical barrier of the host epidermis, resulting in fungal entry into the inner host tissues (5). Several studies have reported the significant role of environmental factors, including nutrient availability, light, humidity, and temperature, on conidial germination (1, 6–8). Variation in the lifestyles of fungi also influences the stages involved in conidial germination and appressorium formation (5, 9–11). Elucidating gene expression patterns involved in each stage is important for managing fungal growth on hosts.

Comparative transcriptomics is a valuable approach to gaining a deeper understanding of changes in gene expression patterns associated with alternate lifestyles among related fungal species. The technique provids information on the genetic basis of specific adaptations, such as those related to pathogenicity, symbiosis, and response to environmental stress. Additionally, comparative transcriptomics reveals how gene expression patterns have diverged or remained conserved across related species, providing insights into the molecular mechanisms of evolution and adaptation (12–15). Previously, we applied the technique to identify the molecular mechanisms involved in conidial germination of *Fusarium graminearum* (Order Hypocreales) and *Magnaporthe oryzae* (Order *Magnaporthales*)*,* both pathogenic to hosts in the Poaceae. The comparative transcriptomic approach revealed that the two species displayed similar gene expression patterns for the pre-penetration stages (16). However, substantial divergence in expression patterns between the two species was observed during appressorium formation. The study provided information on gene function associated with pathogenesis in fungal species of Orders *Hypocreale* and *Magnaporthales*. In the present study, *F. graminearum* and *Metarhizium anisopliae* were compared*,* having different lifestyles, but both belonging to the Order *Hypocreales*.

Mutualistic and pathogenic fungi show distinct spore germination mechanisms on hosts, regulated by the activation of specific gene sets, due to their contrasting ecological roles and interactions with their plant hosts. Phytopathogenic fungi often express virulence factors and may employ strategies to weaken the host’s defense system, including producing enzymes, toxic metabolites, or invasive structures to breach the host’s barriers (17–22). Mutualistic fungi have evolved mechanisms to recognize and interact with their compatible hosts, often exhibiting a relatively passive colonization process where both partners benefit or only one partner benefits without harming the other (23, 24). Here, we analyze and compare the transcriptomic profiles of *F. graminearum* and *M. anisopliae* under two nutrient conditions (on medium and on the host) across four phases of conidial germination. *F. graminearum* is a mycotoxigenic phytopathogen that causes severe economic losses to cereal growers worldwide as the causal agent of Fusarium Head Blight (FHB) affecting wheat, barley, oats, and maize (25, 26). The FHB disease cycle is initiated when spores (conidia and/or ascospores) are deposited on the spikelets of a host plant, germinate, and form infection structures (27). *M. anisopliae* is an endophytic fungus that primarily colonizes root tissues of both monocots and dicot plants without causing any disease symptoms (28–30). In addition, *M. anisopliae* is a well-known insect pathogen with a wide host range (31, 32) and has the ability to transfer nitrogen from infected insects to plant hosts in exchange for carbon, thereby forming mutualistic associations with plant hosts (30, 33). While the process of conidial germination and subsequent penetration of insect hosts is well documented, there is little information on the gene expression changes during the initial stages of interaction with plants. Here, we used transcriptome analysis of the spore germination stages of *M. anisopliae* and compared them to the same stages in *F. graminearum*, to understand the adaptive mechanisms employed by each species during initial colonization on the host. *F. graminearum* is phylogenetically closely related to *M. anisopliae*. Moreover, *F. graminearum* possesses a well-annotated genome, and its infection process has been extensively characterized, making it a valuable reference for comparative analysis. A comparative overview of the taxonomic classification, lifestyle, and genomic features of *F. graminearum* and *M. anisopliae* is given in Table 1. To follow host infection, barley leaf sheaths were inoculated with conidia from *F. graminearum,* and barley roots inoculated with *M. anisopliae* were studied. We focused on the expression profile of the genes encoding carbohydrate-active enzymes, specialized metabolites, and putative effectors across four stages of conidial germination, as these genes play critical roles in early fungal colonization on plants.

**TABLE 1:** Comparison between *F. graminearum* and *M. anisopliae*

	*F. graminearum*	*M. anisopliae*
Class	Sordariomycetes	Sordariomycetes
Order	Hypocreales	Hypocreales
Family	Nectriaceae	Clavicipitaceae
Lifestyle/ pathogenicity	Soil saprophyte; plant pathogen; causes Fusarium Head Blight in cereals, stalk rot and ear rot in maize	Soil saprophyte; insect pathogen; endophyte; causes green muscardine disease in insects
Host	Major pathogen of cereal crops; infects both monocots and dicot plants	Major pathogen of insects; colonizes endophytically both monocot and dicot plants
Genome size	~36 Mb	~38 Mb
No. of predicted genes	~14,000	~11,000

## MATERIALS AND METHODS

### Fungal isolates and experimental design

Wild-type (WT) *F. graminearum* (PH-1, NRRL31084) and *M. anisopliae* (ARSEF 549) cultures were routinely grown on V8 agar at room temperature (RT, 22–24°C) and Potato Dextrose Agar (PDA, Neogen, Lansing, MI, USA) at 24°C, respectively. For both cultures, colonized agar blocks were stored long-term in 35% glycerol at −80°C. Conidia were generated for *F. graminearum* in carboxymethyl cellulose broth (CMC) in shaker culture in the dark for 5 days and then separated from hyphae by filtration through a double layer of Miracloth (Sigma-Aldrich Inc, St. Louis, MO, USA). Conidia were collected by centrifugation, rinsed three times in sterile distilled water (dH_2_O), and resuspended in sterile dH_2_O to a final concentration of 1 × 10^6^ conidia/mL. For *M. anisopliae*, fresh conidia were produced in cultures grown in the dark on PDA for 10–12 days. Conidia were collected by flooding the plates with sterile 0.01% Triton X-100 in dH_2_O, rinsed, and adjusted to 1 × 10^6^ conidia/mL as for *F. graminearum*. For germination studies, PDA, which supports the conidial germination of both species, was used as a common garden environment. A 200 µL aliquot of the conidial suspension of each species was inoculated onto a sterile cellophane sheet (Research Products International, Mount Prospect, IL, USA) overlaid on PDA in a Petri dish (60 × 15 mm). Petri dishes containing inoculated sheets were then incubated at RT in continuous light (14-Watt, 6,500 K, T12 white) till the conidia reached the appropriate germination stage. For *F. graminearum,* cellophane sheets from 30 plates were collected for each of three replicates, and total RNA was extracted from each germination stage. For *M. anisopliae,* cellophane sheets from 60 plates were pooled for RNA extraction for each replicate of each germination stage.

Barley leaf sheaths or roots were used to generate the transcriptome of conidial germination of the two species on the host. Leaf sheaths from 3-week-old barley seedlings were used to generate the transcriptomic profile of *F. graminearum* during initial pathogenesis. *Hordeum vulgare*, cultivar “Stander,” was grown in Suremix medium (Michigan Grower Products, Inc., Galesburg, MI, USA) under greenhouse conditions (22°C with a photoperiod of 16:8 h light:dark cycle). A conidial suspension of *F. graminearum* was prepared as described above, except the final concentration was adjusted to 2.5 × 10^6^ conidia/mL. To monitor germination stages, a 50 µL aliquot of conidial suspension was inoculated onto detached barley leaf sheaths (4–5 cm long) placed in a Petri dish lined with a moist filter paper to maintain humidity. The culture dishes were then incubated at RT in continuous light. One replicate consisted of 20 barley leaf sheaths inoculated with *F. graminearum*, collected for each germination stage for total RNA extraction. Three independent biological replicates were prepared for each germination stage of *F. graminearum* and *M. anisopliae*. Barley roots (cultivar “Stander”) were used to examine conidial developmental stages on the host. Surface-sterilized barley seeds were germinated on 1% water agar. Germinated barley seedlings (4–5 days old) were transferred to a Petri dish, and 200 µL of the conidial suspension (2.5 × 10^6^ conidia/mL) was spread over the roots of each seedling and incubated at 24°C. The roots of five barley seedlings were collected for each stage, and RNA was extracted. Each fungal germination stage on barley or PDA was examined and documented with light microscopy to confirm that at least 80% of the conidia reached the designated germination stage. Chlorazol black E and trypan blue were used to stain the *F. graminearum* and *M. anisopliae* cells on the host, respectively (16).

### cDNA library preparation and RNA-sequencing

Total RNA of *F. graminearum* and *M. anisopliae* grown on cellophane membranes and on host tissue was isolated using Trizol reagent (Thermo Fisher Scientific, Waltham, MA, USA), as per manufacturer’s instructions. Harvested RNA was treated with RNase-Free DNase (Qiagen, Hilden, Germany), purified further using RNA Clean and Concentrator - 5 (ZymoResearch, Irvine, CA, USA), and quantified using the Qubit Fluorometer (Invitrogen, Carlsbad, CA, USA). The integrity and quality of RNA samples were analyzed on High Sensitivity RNA ScreenTape (Agilent Technologies Inc., Santa Clara, CA, USA). The samples measuring with RNA integrity numbers greater than seven were submitted for RNA sequencing at the Research Technology Support Facility (RTSF) Genomics Core at Michigan State University (East Lansing, MI, USA, https://rtsf.natsci.msu.edu/). The cDNA libraries were prepared using the Illumina Stranded mRNA Library Prep Kit (Illumina, Inc., San Diego, CA, USA), and library sequencing was performed using either the HiSeq4000 (single-end, 50 bp) or the NovaSeq6000 (single-end, 100 bp) Illumina (Illumina, Inc., San Diego, CA, USA) platform. Sequences were stored in FASTQ format.

### RNA-seq and differential expression analysis

The quality of the RNA-sequencing raw reads of transcriptome from conidial germination stages of *F. graminearum* and *M. anisopliae* was assessed with FastQC, and the low-quality reads were removed using the read filtering tool, Trimmomatic, Version 0.39 (34). The 3′ ends of NovaSeq6000 single-end 100 bp reads were trimmed to 50 bp reads (keeping the first 50 bp) using Trimmomatic Version 0.39. The reference genome assemblies of *F. graminearum* (35) and *M. anisopliae* (36) were obtained from EnsemblFungi (http://fungi.ensembl.org/). The trimmed reads were mapped to the indexed *F. graminearum* or *M. anisopliae* reference sequences using HISAT 2.2.1 (37). The HISAT alignments in SAM format were then sorted and converted to BAM format using SAM tools (38). Count files were then generated using HTSeq Version 0.11.1 (39). Gene expression patterns in germination Stages 2, 3, and 4 were compared to Stage 1. The DESeq2 package Version 1.30.1 in R was used to normalize the read counts and to perform differential expression analysis. Heatmaps were generated using pheatmap 1.0.12, and row *z*-scores were used to scale the expression between samples. For each species, the Differentially Expressed Genes (DEGs) for germination Stages 2, 3, and 4 compared to Stage 1 were identified using DESeq2. The genes with Log_2_ Fold Change (LFC) ≥2 and adjusted *P*-values (p-adj) ≤0.01 were designated as upregulated, and the genes with LFC ≤ −2 with p-adj ≤0.01 were designated as down-regulated. To further assess the variability among biological replicates, PCA was performed on the rlog-transformed values of transcriptome data of PDA and *in planta* samples.

### Annotation and databases

Carbohydrate-active enzymes (CAZymes) of *F. graminearum* and *M. anisopliae* were predicted using the dbCAN2 meta server (https://bcb.unl.edu/dbCAN2/index.php) HMMER: dbCAN, DIAMOND: CAZy, and HMMER: dbCAN-sub packages (40). Proteins were predicted to be CAZymes when at least two packages of the dbCAN meta server identified a query protein as a CAZyme, and these predicted CAZyme proteins were selected for further analysis. The dbCAN2 metaserver classified CAZymes according to their activity, such as glycoside hydrolases (GH; enzymes involved in the hydrolysis of glycosidic bonds), glycoside transferases (GT; enzymes involved in the formation of glycosidic bonds), polysaccharide lyases (PL, enzymes involved in the non-hydrolytic cleavage of glycosidic bonds), carbohydrate esterases (CE; enzymes involved in the hydrolysis of carbohydrate esters), and auxiliary activities (AA; redox enzymes that act in conjunction with CAZymes). In addition, a non-catalytic class of CAZymes, carbohydrate binding modules (CBM; adhesion to carbohydrates), has also been described (http://www.cazy.org). Specialized metabolite gene clusters were detected in *M. anisopliae* with the antiSMASH 6.0 (fungal version) web server (41). We examined the expression specialized (secondary) metabolite gene clusters that have been identified in the genome of *F. graminearum* previously (42–45). The selected CAZymes and specialized metabolite genes were compared with those identified in previous studies (36, 44) and other databases (http://www.cazy.org and https://mycocosm.jgi.doe.gov/mycocosm/home). EffectorP v 2.0 (46) was used to identify putative effector proteins. SignalP-5.0 was used to detect the presence of signal peptides. Transmembrane domains were detected using DeepTMHMM. We selected the genes with Log_2_ Fold Change (LFC) ≥ 5 and adjusted *P*-values (p-adj) ≤0.01 for CAZyme, specialized metabolite, and effector gene expression analysis. Gene ontology (GO) (47) and Metacyc metabolic pathway analyses (48) were performed using FungiDB (49) with *P*-values < 0.05 considered statistically significant.

## RESULTS

### Transcriptome analysis of conidial germination stages on medium and on the hosts

Spore germination in *F. graminearum* and *M. anisopliae* Stage 1 (fresh spores), Stage 2 (polar growth or germ tube formation), Stage 3 (hyphal extension), and Stage 4 (first hyphal branching in PDA or appressoria formation in the host) were morphologically characterized, and transcriptome profiles were completed on PDA and on barley (Fig. 1). A difference in developmental time course between the growth conditions was not observed in Stages 2 and 3 in *F. graminearum*. At 11 hours (h) on PDA, the first hyphal branches were initiated, while the formation of infection structures on leaf sheaths was observed after 42 h. The majority of *M. anisopliae* conidia reached Stages 2, 3, and 4 in PDA at 10 h, 19 h and 28 h, respectively, whereas on the host, the respective germination stages were reached by 7 h, 15 h, and 26 h.

**Fig 2:**
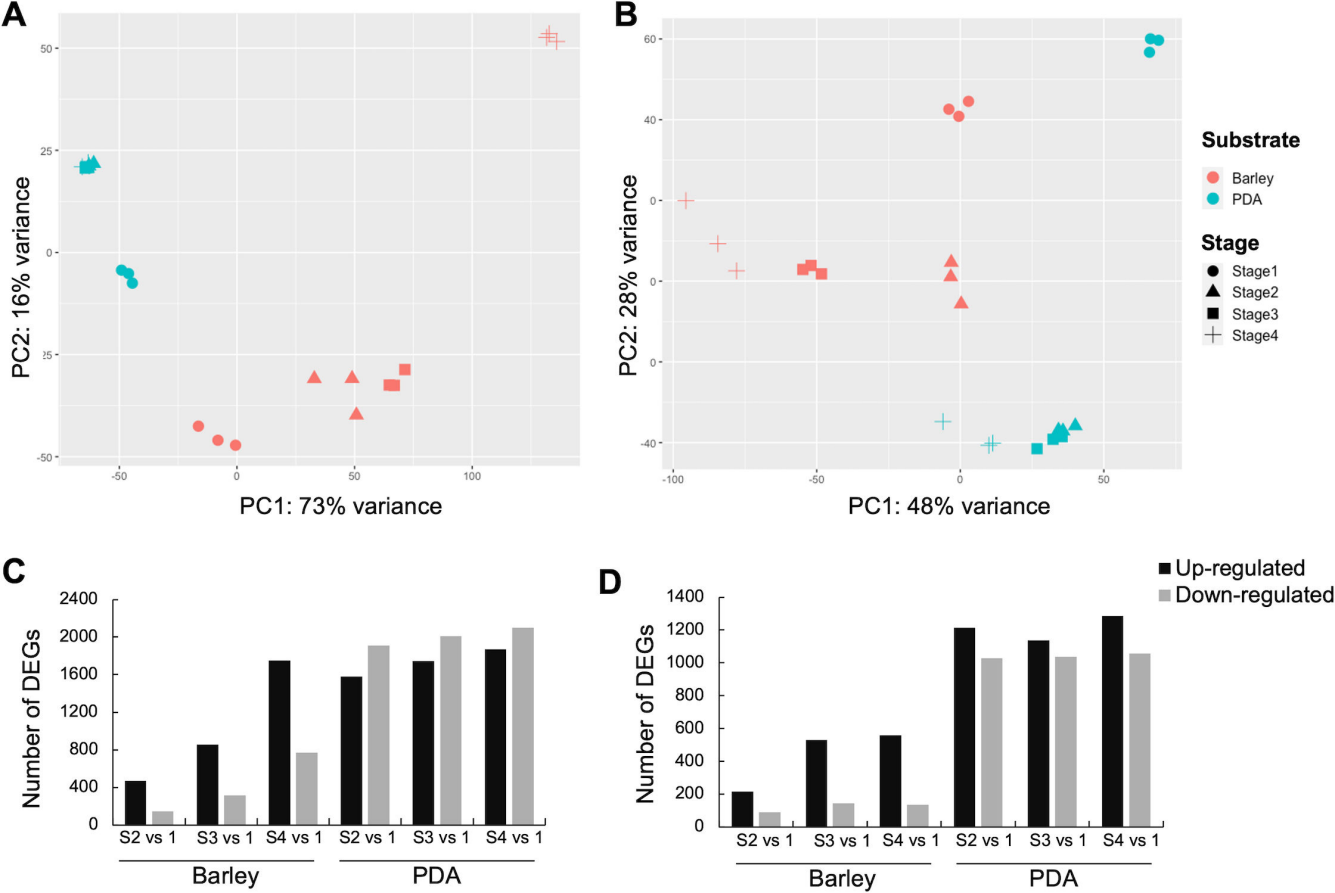
General analysis of the transcriptomes of conidial germination. The principal component analysis of the transcriptome data from germination stages of (**A**) *F. graminearum* and (**B**) *M. anisopliae* on barley and PDA. The number of up- and down-regulated differentially expressed genes (DEGs) of (**C**) *F. graminearum* and (**D**) *M. anisopliae*.

**Fig 3:**
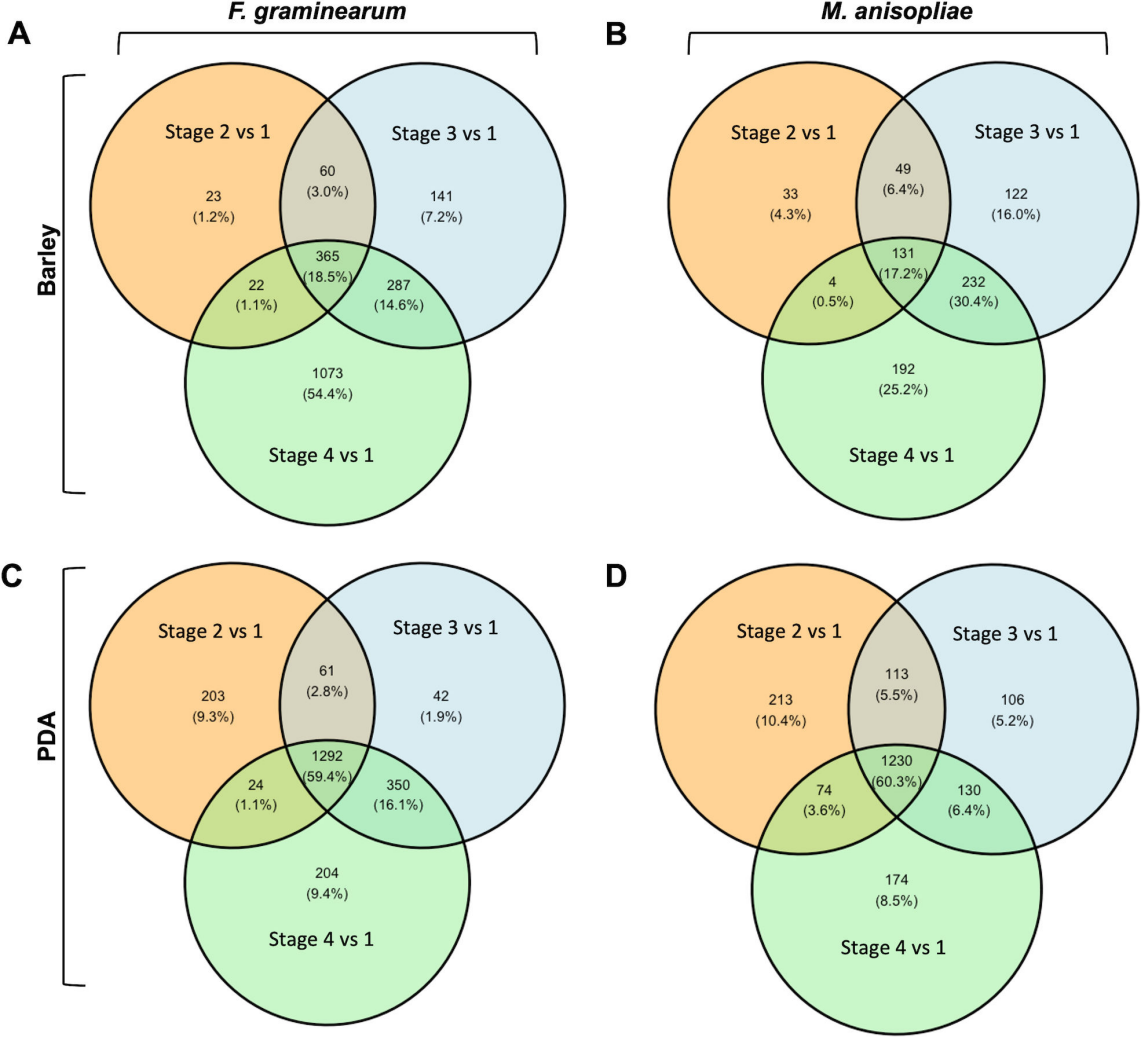
Shared and unique up-regulated DEGs (LFC ≥2 and adjusted p-adj ≤0.01) in the germination stages of *F. graminearum* and *M. anisopliae* on barley and PDA. (**A**) *F. graminearum* on barley; (**B**) *M. anisopliae* on barley; (**C**) *F. graminearum* on PDA; and (**D**) *M. anisopliae* on PDA.

The analysis of the transcriptomes of the conidial germination stages showed that the appressorium stages in *F. graminearum* and *M. anisopliae* were transcriptionally divergent compared to other germination stages. Principal Component Analysis (PCA) plots revealed that germination stages of *F. graminearum* (Fig. 2A) and *M. anisopliae* (Fig. 2B) were grouped separately on PDA and on barley, displaying the differences in transcriptome profiles between these artificial and natural conditions. Clustering also revealed similarity of gene expression profiles among biologically independent replicates, indicating a high level of replicability for the samples. The DEGs in *F. graminearum* and in *M. anisopliae* for all germination stages under both conditions are presented in Tables S1 and S2, respectively. The numbers of downregulated DEGs for all stages of *F. graminearum* grown on PDA were higher compared to those grown on barley. A higher number of upregulated DEGs compared to downregulated DEGs were observed specifically during the appressorium stage on the host (Fig. 2C; Fig. S1A through F), suggesting a dynamic gene activation process during host colonization. For *M. anisopliae,* the number of upregulated DEGs was higher than the number of downregulated DEGs for all stages in both nutritional conditions (Fig. 2D; Fig. S2A through F). The numbers of shared and unique upregulated DEGs among the germination stages on PDA and barley (Fig. 3A through D) demonstrated that the appressorium stage is transcriptionally highly divergent from the rest of the germination stages in both species. We performed k-means clustering of DEGs to identify the top 100 genes that show variation in expression between two nutritional conditions. The appressorium stage showed a higher level of variation in the expression of genes in both species. In *F. graminearum,* the variable genes with higher expression levels were associated with specialized metabolite biosynthesis (Cluster 1) and carbohydrate metabolism (Cluster 3) (Fig. S3A). In *M. anisopliae*, cluster 4 contains the highly variable up-regulated genes for the appressorium stage that were associated with fatty acid metabolism, specialized metabolite biosynthesis, and effector protein encoding genes (Fig. S3B).

### Gene ontology and metabolic pathway analysis of upregulated DEGs

Gene Ontology (GO) and metabolic pathway analyses of *F. graminearum* (Fig. S4; Tables S3 and S5) and *M. anisopliae* (Fig. S5; Tables S4 and S6) revealed that during germination on the host, more carbohydrate breakdown and utilization genes were upregulated compared to the expression of the same genes during germination on PDA, indicating a differential upregulation of CAZymes on the host in two fungal species. For all germination stages in *F. graminearum* in barley, the most significant enriched molecular function is “hydrolase activity.” Hydrolyzing the glycosyl bonds suggests that a high number of CAZymes are differentially upregulated during germination on the host. In *M. anisopliae*, “carbohydrate metabolic process,” “isomerase activity,” “glycolytic process,” and “pyruvate metabolic process” appeared among the highly significant GO terms enriched for all the germination stages in the host. However, in *F. graminearum*, more carbohydrate breakdown and utilization genes were upregulated during conidial germination on the host, than in *M. anisopliae*, particularly those targeting plant cell walls. The enrichment of GO terms for “secondary metabolite biosynthesis” during Stage 4 on the host supports a role for specialized metabolites during early stages of infection in *F. graminearum* (Fig. S4C).

Metabolic pathway analysis revealed that plant-specific polysaccharide degrading pathways were the most differentially upregulated genes involved in the *F. graminearum*-barley association that were common for all the germination stages. These include cellulose degradation II (fungi) (PWY-6788) with the highest significance and highest gene count, followed by (1, 4)-b-D-xylan degradation (PWY-6717), cellulose and hemicellulose degradation (PWY-6784), and pectin degradation II (PWY-7248). Genes related to appressorium development were also upregulated in *F. graminearum* at Stage 4 on the host. Notably, the “o-diquinones biosynthesis” pathway was specifically enriched at this stage, including five tyrosinase-encoding genes (Table S5). Tyrosinases are components of the melanin biosynthesis pathway, where they contribute to both appressorium development and pathogenesis (50). GO enrichment analysis on PDA for both species showed the enrichment of the GO term, “microtubule based movement,” suggesting the movement of organelles and microtubules was more active on the medium than they were during interactions with their natural host (Fig. S4D through F, S5D through F; Table S3 and S4). The top metabolic pathways that were enriched in *M. anisopliae* during germination stages on the host include sucrose biosynthesis (SUCSYN-PWY), trehalose degradation (PWY-2723), glucose and glucose-1-phosphate degradation (glucose1pMETAB-PWY), and superoxide radicals or reactive oxygen species degradation (DETOX1-PWY). Interestingly, metabolic pathway analysis in *M. anisopliae* revealed that genes associated with IAA biosynthesis were upregulated during germination Stages 3 and 4 on barley. IAA biosynthesis genes were enriched in the L-tryptophan degradation pathways (PWY-6307 and TRYPDEG) (Table S6). Common metabolic pathways between the two species for each germination stage were examined (Fig. S6A through C; Table S7). During Stage 4 on the host, genes for degrading plant defense compounds were upregulated in both *F. graminearum* and *M. anisopliae*, suggesting their adaptation for bypassing plant defenses during the appressoria formation (Table S7).

### Differential expression of CAZyme genes

**Fig 4:**
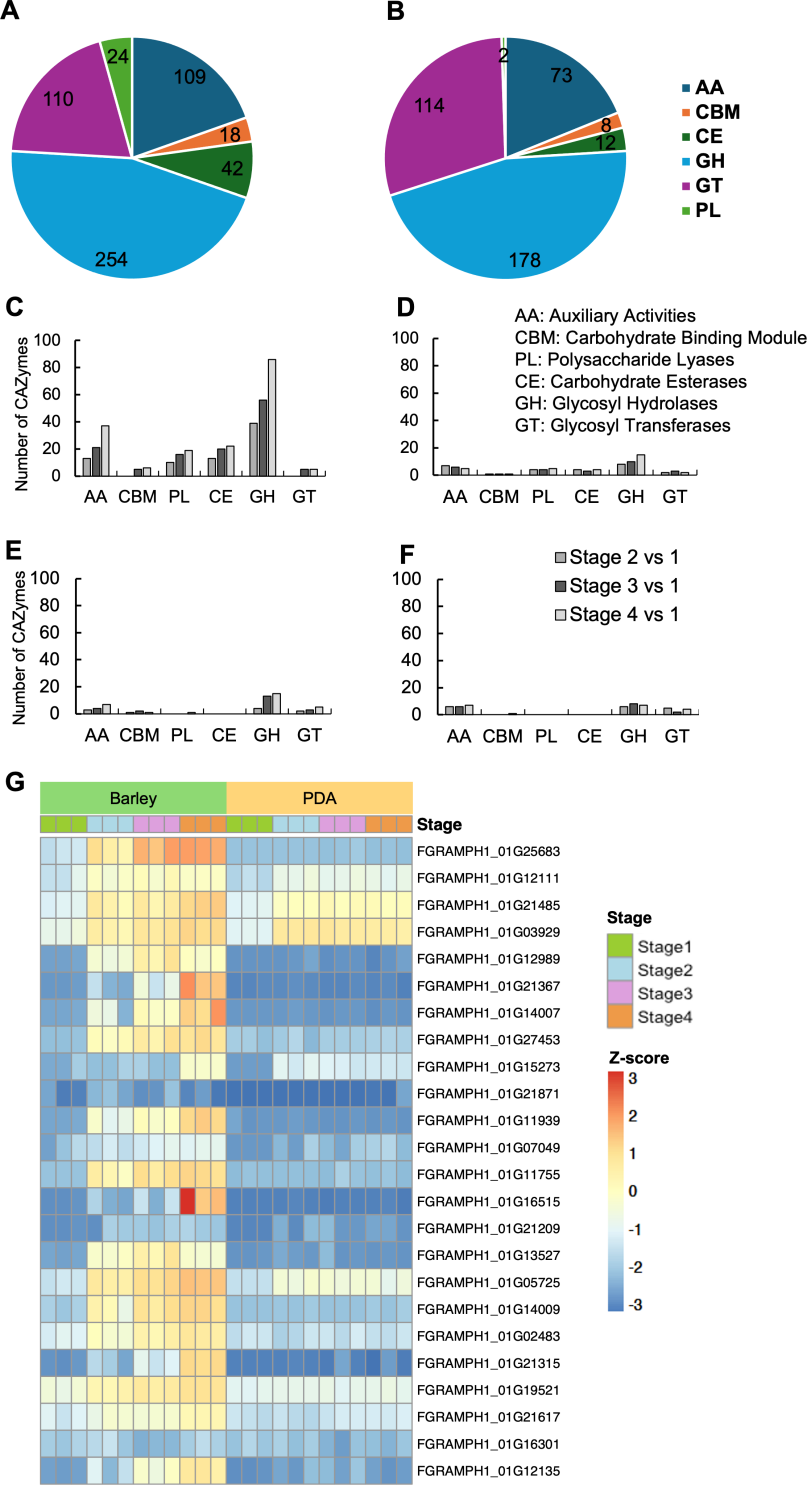
Differential expression of CAZyme genes in the germination stages of *F. graminearum* and *M. anisopliae*. CAZyme classes identified in the genome of (**A**) *F. graminearum* and (**B**) *M. anisopliae*. Number of differentially expressed genes encoding CAZymes during Stages 2, 3, and 4 in (**C**) *F. graminearum* on barley, (**D**) *F. graminearum* on PDA, (**E**) *M. anisopliae* on barley, (**F**) *M. anisopliae* on PDA. (**G**) Expression of polysaccharide lyase (PL) genes in *F. graminearum*. AA, auxiliary activities; CBM, carbohydrate binding module; PL, polysaccharide lyases; CE, carbohydrate esterases; GH, glycosyl hydrolases; GT, glycosyl transferases.

GO enrichment analysis suggests that carbohydrate metabolism is highly active during germination stages of both fungal species on barley with evidence that the *F. graminearum* genome harbors 170 more genes encoding CAZymes than does *M. anisopliae* (Fig. 4A and B) and expression of CAZymes differed substantially between *F. graminearum* and *M. anisopliae* (Fig. 4C through F). *F. graminearum* expressed a higher number of upregulated CAZymes during germination on barley (13.4% [Stage 2], 21.9% [Stage 3], 31.4% [Stage 4]) compared to PDA (4.6% [Stage 2], 4.8% [Stage 3], 5.7% [Stage 4]) (Fig. 4C and D; Table S8), suggesting its ability to penetrate the host tissue and cause necrosis soon after the initiation of infection. In *M. anisopliae,* fewer CAZymes were differentially expressed overall (Fig. 4E and F; Table S9). During germination stages on the host, *M. anisopliae* expressed 2.5%, 5.6%, and 7.4% of CAZymes at Stages 2, 3, and 4, respectively, and the expression levels were 4.3%, 4.1%, and 4.9% at the same respective stages on PDA. However, a 1.5-fold increase in the expression of glycoside hydrolases was observed during germination Stages 3 and 4 on the hosts compared to PDA. These results suggest a critical role for CAZymes in both pathogenic and endophytic species during early stages of colonization on the hosts. *F. graminearum* displayed the upregulation of both chitin synthase and cutinase genes (Table S8), which was not observed in *M. anisopliae*.

Among the PLs identified in *F. graminearum,* 80.3% were differentially upregulated during the appressorium stage on the host (Fig. 4G), suggesting the degradation of plant cell walls to initiate infection. The *M. anisopliae* genome harbors only two PL genes, and one PL gene (MAN_03654) was found differentially upregulated in Stage 4 on the host. *F. graminearum* genome contains numerous genes for pectin-degrading enzymes which are differentially upregulated during germination on the host, especially in the appressorium stage. In *M. anisopliae*, the pectin-degrading enzyme genes were not upregulated during host colonization (Table S9), suggesting a minimal role of pectin-degrading enzymes during the initial stages of host colonization.

### Differential expression of specialized metabolite genes

We observed substantial differences in the expression of specialized metabolite genes between the two species during host colonization. During the appressorium stage in *F. graminearum*, the expression of specialized genes increased significantly, with a 3.4- to 6.8-fold upregulation compared to other germination stages (Table S10). The trichothecene biosynthetic gene cluster was upregulated during the appressorium stage on barley (Fig. S7; Table 2), likely producing trichothecenes in early host colonization events. In *M. anisopliae*, the 58.06% of specialized metabolite genes upregulated during the appressorium stage on the host were the same as upregulated on PDA (Table S11). Interestingly, the metabolic pathway analysis showed that genes associated with tryptophan metabolism were upregulated in Stages 3 and 4 on the host. Tryptophan is a precursor for the biosynthesis of phytohormone, IAA, that regulates various aspects of plant growth and development (51–53). *M. anisopliae* synthesizes indole-3-acetic acid (IAA), through tryptophan-dependent pathways (54). Further analysis revealed that the candidate genes encoding enzymes associated with IAA biosynthesis, including anthranilate phosphoribosyltransferase, nitrilase, and flavin monooxygenase, were upregulated in *M. anisopliae* in Stage 4 on the host (Table S12), supporting a role of IAA metabolism in the interaction between *M. anisopliae* and the host.

**TABLE 2:** The major specialized metabolite gene clusters upregulated in *F. graminearum* during germination stages on the host and PDA^*a*^

	Barley			PDA		
	Stage 2 vs 1	Stage 3 vs 1	Stage 4 vs 1	Stage 2 vs 1	Stage 3 vs 1	Stage 4 vs 1
Orcinol/orsellinic acid	2	3	4	4	4	4
Triacetylfusarinine	0	0	2	0	0	0
Trichothecene	1	2	11	1	2	1
Butenolide	0	1	1	0	0	0
Precursor of insoluble perithecial pigment	1	1	1	0	0	0
Malonichrome	0	0	3	0	0	0
Decalin-containing diterpenoid pyrones	0	0	7	0	0	0

^*a*^ The specialized metabolite genes with LFC ≥5 and *p-*adj ≤0.01 were selected for the analysis, and the table shows the number of genes upregulated in each biosynthetic gene cluster.

### Differential expression of effector genes

**Fig 5:**
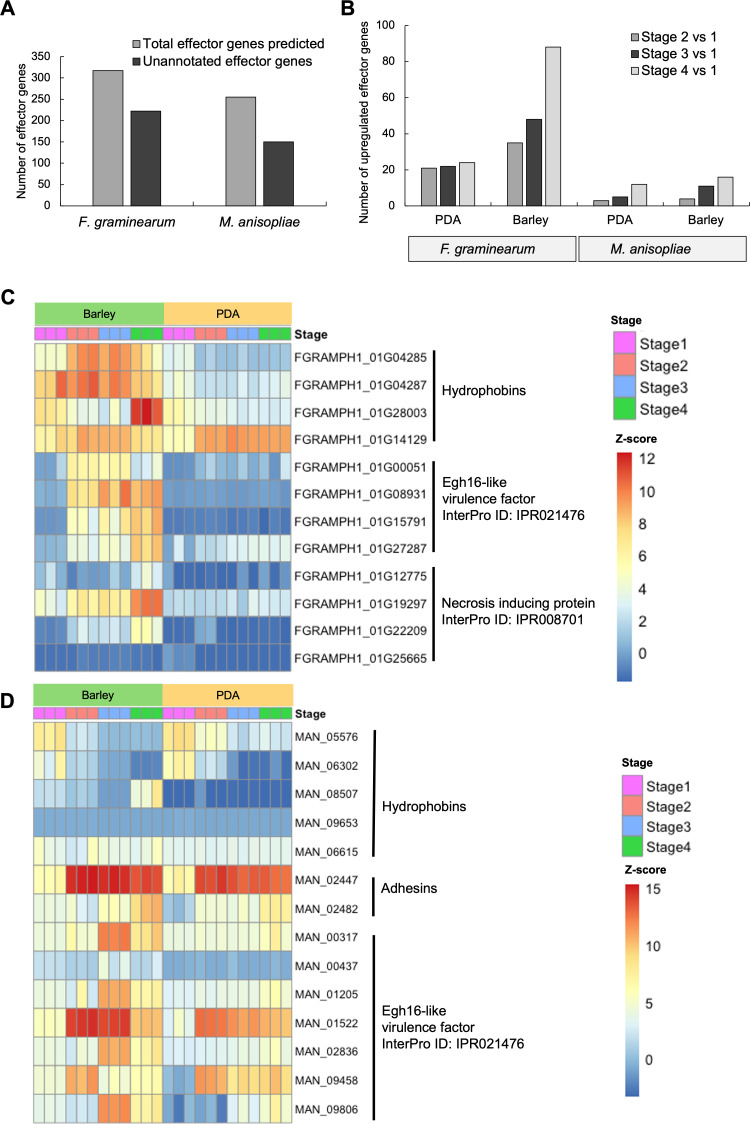
Differential expression of effector genes in the germination stages of *F. graminearum* and *M. anisopliae*. (**A**) Comparison of putative effector genes identified in the genome of *F. graminearum* and *M. anisopliae*. (**B**) Number of upregulated effector genes during germination stages in *F. graminearum* and *M. anisopliae* on PDA and barley. Expression of pathogenicity-related genes in germination stages on PDA and barley in (**C**) *F. graminearum* and (**D**) *M. anisopliae*. Heatmap color range represents high to low expression levels where red represents higher expression and blue represents lower expression.

Our analysis indicates that the genome of *F. graminearum* encodes more secreted putative effector genes in comparison to that of *M. anisopliae* (Fig. 5A). In both *F. graminearum* and *M. anisopliae,* the majority of these putative effectors are annotated as putative small-secreted cysteine-rich proteins or proteins with unknown function. Our results indicate that genes encoding secreted putative effectors are upregulated during the appressorium stages in both species, with *F. graminearum* expressing more effectors overall (Fig. 5B; Tables S13 and S14). Genes encoding putative effectors including AA1-like domain-containing proteins, LysM domain-containing proteins, and the cell wall mannoprotein 1 domain proteins were upregulated in both species during appressorium formation, emphasizing the pivotal role of secreted effector proteins in the early stages of plant colonization for both species. *M. anisopliae* upregulated putative effectors included a calcium channel inhibitor (Killer toxin Kp4) and a proteinase inhibitor I1 (Kazal domain), both upregulated during the appressorium stage indicating roles in modulating host immune responses during pathogen germination on the host. Besides the predicted effector genes, we also examined the expression of hydrophobin genes, adhesin genes, genes encoding proteins containing necrosis-inducing protein domains (IPR008701), and genes encoding proteins with Egh 16-like virulence factor domains (IPR021476), all of which have been reported in other plant-colonizing as pathogenicity-related genes. Our analysis suggests that the upregulation of hydrophobins during germination stages on the host indicates its role as a virulence factor during initial colonization of *F. graminearum* (Fig. 5C). Additionally, the genes encoding proteins with Egh16-like domains, associated with some virulence factors, were upregulated during the appressorium stage in *F. graminearum*, a key stage in the infection process. However, in *M. anisopliae*, these genes were upregulated during Stages 2 and 3 (Fig. 5D), when the fungal conidia were beginning to germinate, but their expression decreased as the fungus progressed to the appressorium stage.

## DISCUSSION

We compared genome-wide expression of conidial germination stages in pathogenic vs endophytic fungi on barley, using *F. graminearum* and *M. anisopliae*. During the appressorium stage, both fungi exhibited a significant transcriptomic shift compared to other germination stages, highlighting the unique transcriptional changes associated with appressorium development. While *F. graminearum* exhibited a more aggressive colonization strategy, with extensive CAZyme and specialized metabolite involvement, *M. anisopliae* adopted a more subtle approach by expressing phytohormone biosynthetic genes and fewer CAZyme genes, to establish a symbiotic relationship with its host. Both fungi showed the upregulation of the genes encoding the LysM effector, suggesting the importance of effectors in modulating host immune responses for successful host colonization.

Here, we documented the formation of appressoria by *M. anisopliae* during germination on its host (on barley roots). Previously, among species of *Metarhizium,* only *M. robertsii* had been reported to produce appressorium-like structures, with the presence of swelling at the germling tip on *Arabidopsis* roots 1 day post inoculation (55). We observed the formation of similar structures in *M. anisopliae* during germination on barley roots. Spore germination occurred earlier on the barley root surface in *M. anisopliae* compared to growth on PDA, with the appressoria forming 26 h after inoculation of the roots. Phytohormones, including strigolactones, IAA, and gibberellic acid, have been shown to influence the maintenance of *Metarhizium*-plant associations (56, 57) and have been found to induce conidial germination and hyphal growth in *M. guizhouense* when assayed on media amended with phytohormones (58). Similar results were also reported in mycorrhizal fungi, which exhibit extensive hyphal branching in the proximity of host roots, suggesting a role for plant metabolites in promoting fungal conidial germination and growth during beneficial fungal-plant interactions (59, 60), consistent with our results.

The *F. graminearum* genome harbors more CAZyme-encoding genes than the genome of *M. anisopliae* (Fig. 4A and B). The abundance of CAZymes in *F. graminearum*, together with the higher expression levels of these genes during interactions with the host, provides supporting evidence for the enhanced capacity of *F. graminearum* to penetrate the plant host and initiate disease. In contrast, *M. anisopliae* has limited the expression of genes for plant cell wall-degrading enzymes during initial colonization, reflecting a less aggressive approach for establishment in the host, as would be expected for an endophyte. In general, fungal colonization is initiated when conidia first adhere to and then breach the cuticle. Cutinases act as surface-sensing virulence factors and are crucial for establishing infection in above-ground plant parts (61). Cutinase has been reported as essential in disease development in phytopathogenic fungi including *Botryosphaeria dothidea* (62)*, F. sacchari* (63)*, Magnaporthe oryzae* (64), and *Sclerotinia sclerotiorum* (65), and cutinase genes are upregulated in *F. graminearum* during appressorium formation. We found a lower number of cutinase and polysaccharide lyase genes in the *M. anisopliae* genome than in the *F. graminearum* genome. Additionally, we documented a low germination rate for *M. anisopliae* when inoculated onto barley leaf sheaths (data not shown) when compared to the barley roots. The limited expression of CAZyme genes in *M. anisopliae* allows it to enter the host while minimizing damage, which is consistent with its ability to colonize root cortical cells and intracellular spaces with limited impact on plant health (29).

Our data show that multiple specialized metabolite gene clusters are upregulated during appressorium formation in *F. graminearum* compared to *M. anisopliae*. However, the metabolic pathway analysis of upregulated genes of *M. anisopliae* on the host showed that the tryptophan metabolic pathway was more highly expressed in the appressorium stage. Tryptophan metabolism is linked to the IAA synthesis, and we found that the candidate genes involved in the synthesis of IAA were upregulated in the appressorium stage. *Metarhizium* spp. have been reported to promote root hair growth promotion within 1–2 days of colonization of the host (29). Previous studies showed that IAA influences the growth and architecture of roots, which are important for nutrient and water uptake (66, 67). The ability of *M. anisopliae* to induce root growth in plants through IAA-dependent mechanisms (54) demonstrates the intricate and multifaceted relationship that exists between this endophyte and its plant host. This difference suggests how *F. graminearum* uses a wider range of specialized metabolites for aggressive plant colonization, while *M. anisopliae* employs a phytohormone-based strategy to interact with its plant host.

Our results indicate that genes encoding secreted putative effectors are upregulated during the appressorium stage for both species, emphasizing the pivotal role of secreted effector proteins in the early stages of plant colonization, regardless of the lifestyle of the fungus. Secreted effectors are generally described as cysteine-rich proteins with a signal peptide for secretion and have no transmembrane domain, used by plant-colonizing fungi to manipulate the host defense responses (68, 69). However, the number of putative effectors expressed was higher in the phytopathogen, *F. graminearum,* compared to *M. anisopliae*, an endophyte and mutualist. We found that putative effector genes, including LysM protein coding genes reported in other plant colonizing fungi (70–72), were upregulated in both *M. anisopliae* and *F. graminearum* during colonization of the host. The LysM domain in the effectors suppresses the chitin-induced plant defense response and has been identified previously in other plant colonizing fungi as modulating host immunity (73). The role of LysM proteins in modulating defense responses in plant hosts has been described in *Botryosphaeria dothidea* (74), *Trichoderma atroviride* (75), and *Verticillium dahliae* (76). The deletion of the *lysm2* gene reduced the ability of beneficial plant root colonizer *Clonostachys rosea* to colonize wheat roots (77). The two LysM proteins, ChELP1 and ChELP2, in the anthracnose fungus *Colletrotrichum higginsianum,* have been shown to be essential for fungal attachment, appressorium function, and suppression of chitin-triggered immunity of hosts (78). The differential upregulation of putative LysM type effectors in both *M. anisopliae* and *F. graminearum* during appressorium formation suggests that suppressing chitin-triggered immunity in plant hosts is an important step during plant colonization and is an evolutionarily conserved trait in both pathogenic and mutualistic/endophytic fungal species.

Our analysis suggests that virulence-associated genes are important for both *F. graminearum* and *M. anisopliae* to initiate interactions with plant hosts. For instance, Egh16-like protein-coding genes, previously reported in both phytopathogenic and entomopathogenic fungi as virulence factors, were upregulated in both *F. graminearum* and *M. anisopliae* during *in planta* growth. In the rice blast fungus, *M. oryzae*, *Egh16*-like genes have been shown to play a role in host penetration and lesion formation (79). Similarly, in *Metarhizium acridum*, a specialist insect pathogen, the deletion of *Magas1*, which encodes a protein containing an Egh16-like virulence factor domain, impaired fungal penetration during topical infection of locusts (80). The involvement of Egh16-like proteins in both plant and insect pathogenesis suggests that these genes may facilitate initial interactions between *M. anisopliae* and its diverse hosts, supporting the dual lifestyle of the fungus as a plant-associated endophyte and an insect pathogen. The specific role of Egh16-like proteins in *M. anisopliae*-plant association requires further investigation. Additionally, we observed increased expression of genes encoding necrosis-inducing proteins during the appressorium stage of *F. graminearum*, suggesting the initiation of the necrotrophic phase of *F. graminearum* even at this early stage of infection. The necrosis-inducing protein family has been reported in plant pathogenic bacteria, oomycetes, and pathogenic fungi, triggering cell death and suppressing defense responses on the host (81). For instance, the necrosis-inducing protein (Nep1) of *F. oxysporum* was implicated in triggering cell death and necrotic spots in *Arabidopsis* (82).

We found major differences in the expression profiles of plant cell wall-degrading enzymes and specialized metabolites between two fungi that play distinct roles during infection of their hosts. *F. graminearum* expresses multiple CAZyme genes and specialized metabolite gene clusters that have been linked to its pathogenic lifestyle, whereas *M. anisopliae* expresses genes associated with phytohormones. Each approach plays a key role in adaptation to a specific lifestyle. Future research should provide an understanding of the functional roles of the CAZymes, effectors, and specialized metabolites identified in this comparative analysis. Additionally, expanding this research to include proteomic and metabolomic approaches will provide a more comprehensive understanding of the underlying biochemical processes of spore germination and adaptive mechanisms employed by these fungi during host interactions. Elucidation of these processes will be critical to developing targeted strategies for managing fungal diseases and for optimizing applications of beneficial fungi.

## Data Availability

The RNA-seq data generated in this work have been deposited in NCBI’s BioProject (https://www.ncbi.nlm.nih.gov/bioproject). The data are accessible through GEO series accession numbers GSE277787 (conidial germination in *F. graminearum*) and GSE277627 (conidial germination in *M. anisopliae*).

## SUPPLEMENTAL MATERIAL

Supplemental material 1 spectrum.00226-25-s0001.xlsx Tables S1 and S2.

Supplemental material 2 spectrum.00226-25-s0002.xlsx Tables S3 and S4.

Supplemental material 3 spectrum.00226-25-s0003.xlsx Tables S5 to S7.

Supplemental material 4 spectrum.00226-25-s0004.xlsx Tables S8 and S9.

Supplemental material 5 spectrum.00226-25-s0005.xlsx Tables S10 and S11.

Supplemental material 6 spectrum.00226-25-s0006.xlsx Tables S13 and S14.

Supplemental figures spectrum.00226-25-s0007.pdf Fig. S1 to S7.

Table S12 spectrum.00226-25-s0008.docx Expression analysis of putative genes involved in IAA biosynthesis in *M. anisopliae* in Stage 4 on the host.

ASM does not own the copyrights to Supplemental Material that may be linked to, or accessed through, an article. The authors have granted ASM a non-exclusive, world-wide license to publish the Supplemental Material files. Please contact the corresponding author directly for reuse.
