# Supplementary material for: Comparative transcriptomics reveal contrasting strategies between a fungal plant pathogen and an endophyte during initial host colonization
Source: Microbiol Spectr. 2025 Jun 12;13(8):e00226-25. doi: 10.1128/spectrum.00226-25 (PMC12323313; doi:10.1128/spectrum.00226-25)
Supplement: Supplemental figures — Fig. S1 to S7. [file spectrum.00226-25-s0007.pdf]

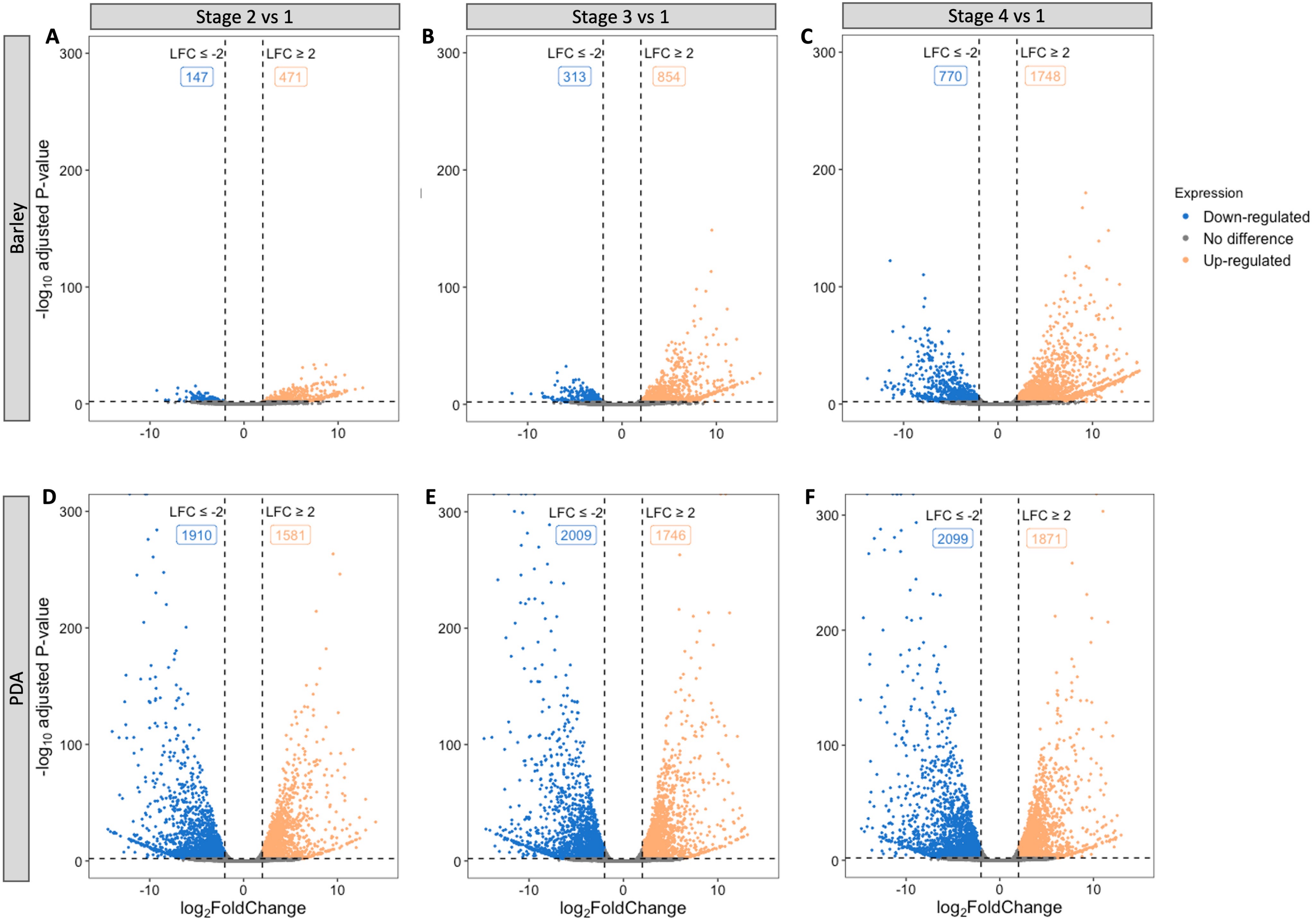

Fig S1. Volcano plots of differentially expressed genes (DEGs) from the transcriptome data of *F. graminearum*. The number of up- or down-regulated DEGs of *F. graminearum* in Stages 2, 3 and 4 vs Stage 1 on (**A-C**) barley and (**D-F**) PDA are shown.

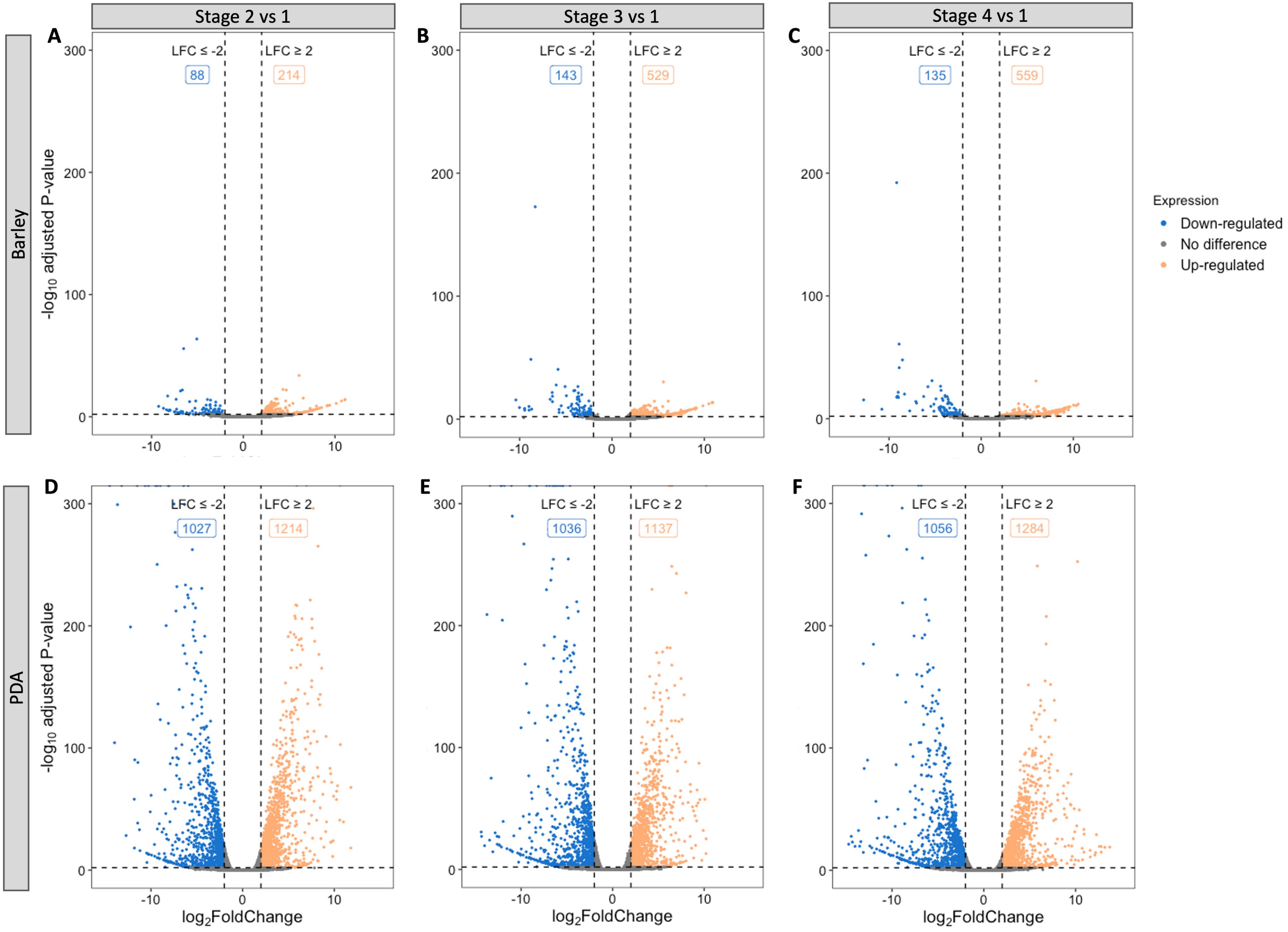

Fig S2. Volcano plots of DEGs from the transcriptome data of *M. anisopliae*. The number of up- or down-regulated DEGs of *M. anisopliae* in Stages 2, 3 and 4 vs Stage 1 on (**A-C**) barley and (**D-F**) PDA are shown.

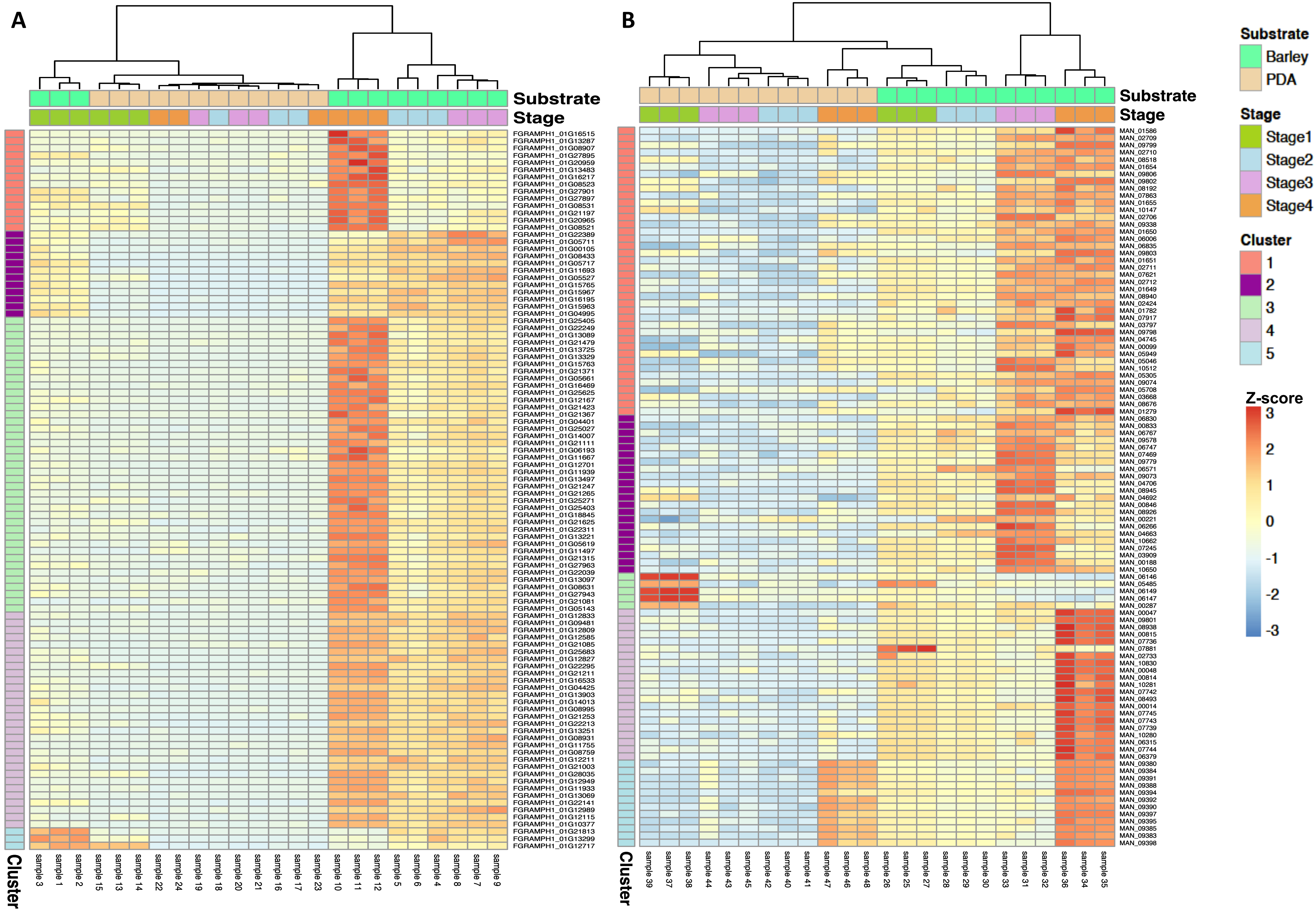

Fig S3. Top 100 genes of (**A**) *F. graminearum*, (**B**) *M. anisopliae* that showed highly variable expression levels during different germination stages on barley and PDA. Genes were grouped into five clusters based on the expression pattern. Heatmap colour range represents high to low expression levels (rlog-transformed normalized counts) where the red represents higher expression and blue represents lower expression.

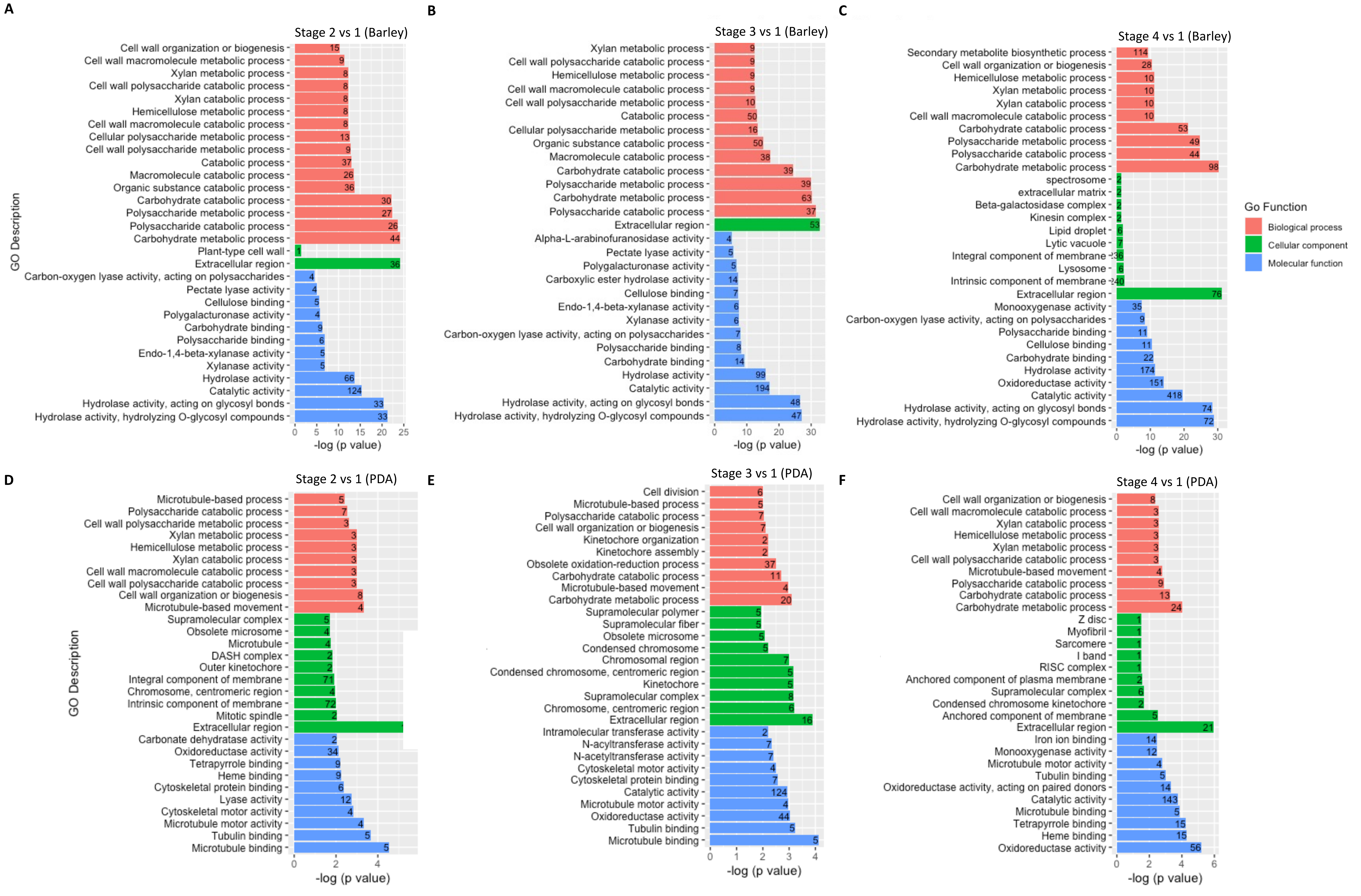

Fig S4. Gene ontology classification of DEGs of *F. graminearum* in different germination stages on (**A-C**) barley and (**D-F**) PDA. The numbers on the bar represent the quantity of upregulated genes (LFC ≥ 5 and adjusted p-adj ≤ 0.01) enriched for each GO term.

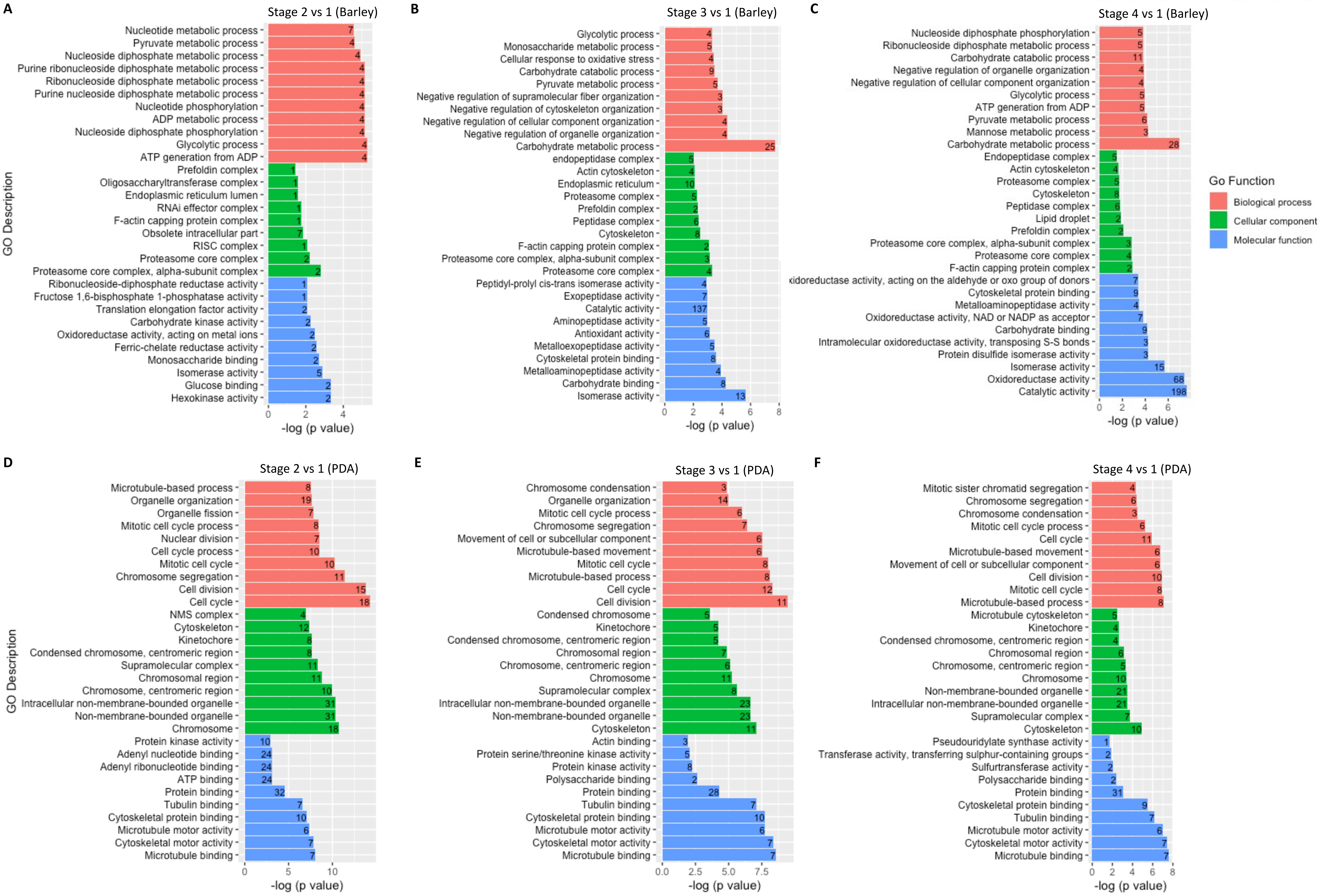

Fig S5. Gene ontology classification of DEGs of *M. anisopliae* in different germination stages on (**A-C**) barley and (**D-F**) PDA. The numbers on the bar represent the quantity of upregulated genes (LFC ≥ 5 and adjusted p-adj ≤ 0.01) enriched for each GO term.

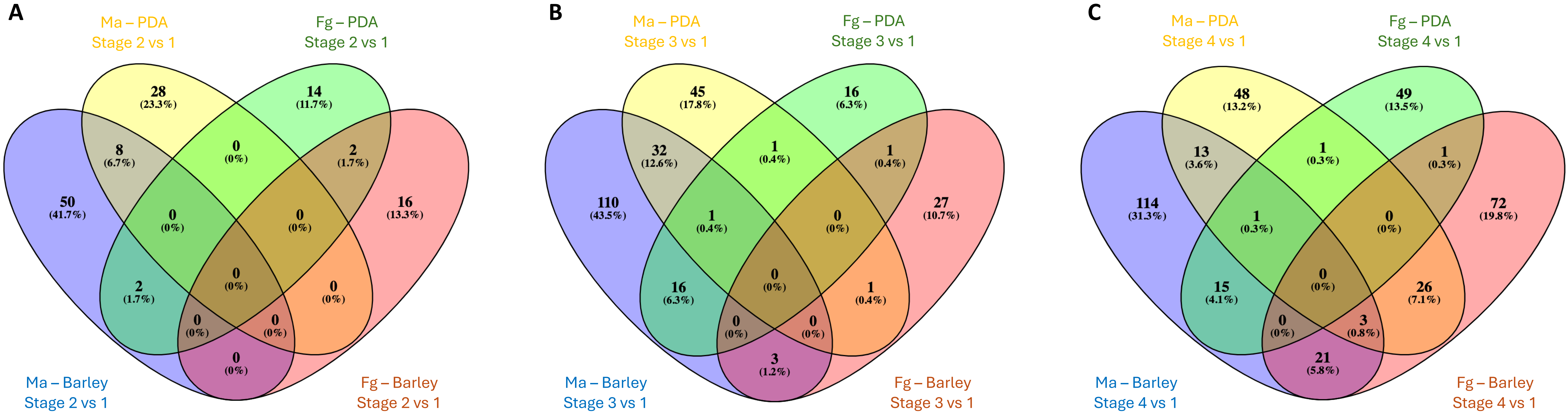

Fig S6. The shared metabolic pathways between *F. graminearum* (Fg) and *M. anisopliae* (Ma) for each germination stage on both nutritional conditions. (**A**) Stage 2 vs 1. (**B**) Stage 3 vs 1. (**C**) Stage 4 vs 1.

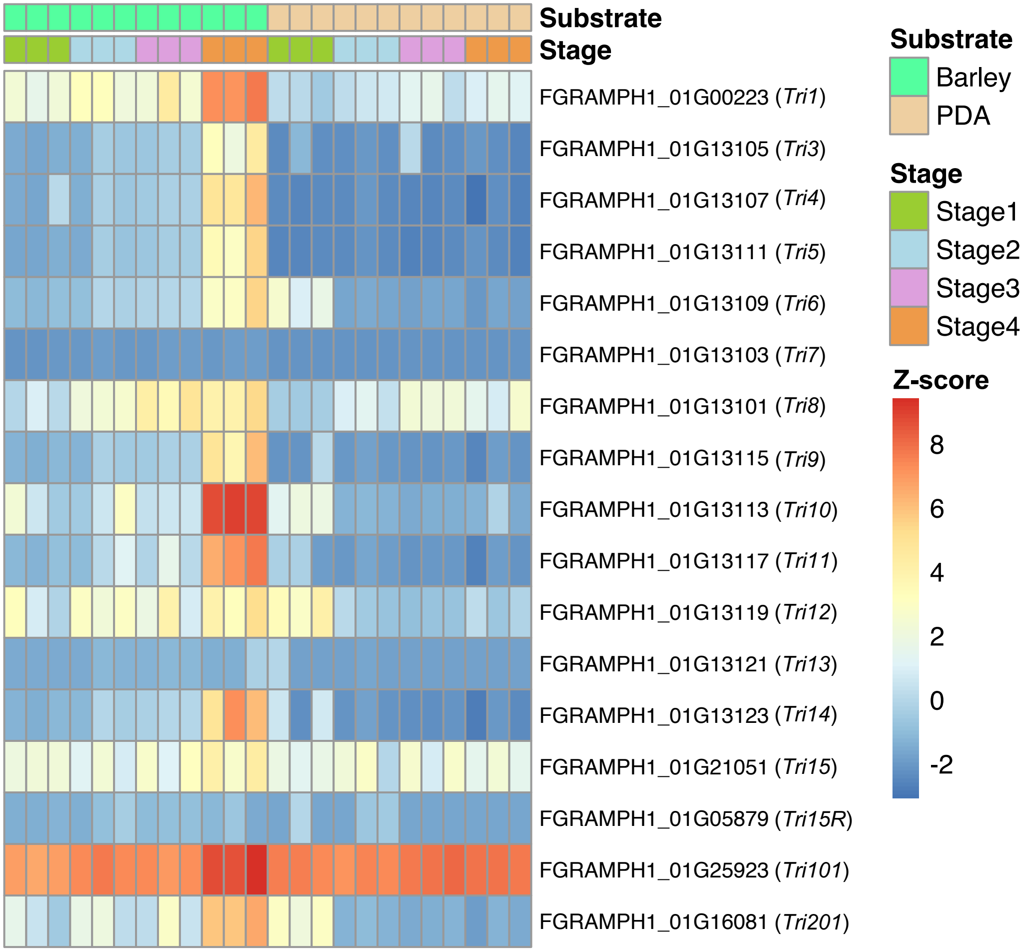

Fig S7. Expression of genes in trichothecene biosynthetic gene cluster during different germination stages on barley and PDA. Heatmap colour range represents high to low expression levels where the red represents higher expression and blue represents lower expression.
