# Supplementary material for: Comparative transcriptomics reveal contrasting strategies between a fungal plant pathogen and an endophyte during initial host colonization
Source: Microbiol Spectr. 2025 Jun 12;13(8):e00226-25. doi: 10.1128/spectrum.00226-25 (PMC12323313; doi:10.1128/spectrum.00226-25)
Supplement: Table S12 — Expression analysis of putative genes involved in IAA biosynthesis in M. anisopliae in Stage 4 on the host. [file spectrum.00226-25-s0008.docx]

**Table S12. Expression analysis of putative genes involved in IAA biosynthesis in *M. anisopliae* in Stage 4 on the host.**

| **Gene** | **Function** | **LFC and p-adj**  **(Stage 4 on the host)** |
| --- | --- | --- |
| Chorismate synthase  (MAN_09314 and MAN_02150) | Chorismate -> Anthranilate | No significant difference |
| Anthranilate phosphoribosyl transferase (MAN_05002) | Anthranilate ---> Tryptophan | 5.84, 0.0001 |
| Nitrilase (MAN_09419)  (**via indole-3-acetaldoxime pathway**) | Indole-3-acetonitrile -> IAA | 7.27, 0.00001 |
| Flavin monooxygenase (MAN_01643)  (**via indole-3-pyruvic acid pathway**) | Indole pyruvate -> IAA | 6.50, 0.0001 |
